# A Systematic Review of Ocular Complications Following Different Types of Covid‐19 Vaccines

**DOI:** 10.1155/jimr/8766021

**Published:** 2025-10-21

**Authors:** Bahareh Ebrahimi, Hossein Kargar Jahromi, Nazanin Shafiei Jahromi, Mina Molayem

**Affiliations:** ^1^ Shiraz Geriatric Research Center, Shiraz University of Medical Sciences, Shiraz, Iran, sums.ac.ir; ^2^ Research Center for Non-Communicable Diseases, Jahrom University of Medical Sciences, Jahrom, Iran, jums.ac.ir; ^3^ Department of Microbiology, Islamic Azad University, Jahrom Branch, Jahrom, Fars, Iran, khuisf.ac.ir

**Keywords:** BNT162 vaccine, ChadOx1 nCoV-19, coronavirus, COVID-19 vaccine, eye diseases, mRNA vaccines

## Abstract

**Introduction:**

More than 6 million deaths from the novel coronavirus, the Coronavirus disease 2019 (COVID‐19) infection, have prompted the development of several prophylactic vaccines of COVID‐19. This systematic review summarizes the ocular complications of various COVID‐19 vaccinations, diseases diagnosed, treatment, and risk factors.

**Methods:**

The search was done in PubMed, Web of Science (WOS), and Google Scholar databases. Manifestations were classified depending on the type of vaccines and the interval between vaccination and the onset of symptoms. Other data such as patients’ age, gender, underlying diseases, and follow‐up data were also extracted.

**Results:**

Initially, 10,242 articles were identified and 76 articles were eligible that among them 107 cases were reported. Ocular complications were diagnosed more often in Pfizer‐BioNTech COVID‐19 vaccine and Oxford–AstraZeneca COVID‐19 vaccine (AstraZeneca) recipients than in others.

**Conclusion:**

This systematic review highlights a wide range of ocular complications reported after COVID‐19 vaccination, the most common of which is uveitis. While most cases were mild and self‐limiting, some involved reactivation of preexisting inflammatory diseases. These findings emphasize the importance of postvaccination ocular surveillance. This is particularly important in individuals with a history of ocular inflammation and suggests a potential immunological mechanism that requires further investigation.

## 1. Introduction

The coronavirus disease 2019 (COVID‐19) pandemic was one of the most severe challenges the world has faced in the last century. The clinical spectrum of COVID‐19 ranges from asymptomatic to critical illness in adults [1–3] and even in children [4, 5]. Various studies have shown that the incidence of ocular symptoms in COVID‐19 patients ranges from 2% to 32%, manifesting at any stage of the disease [6, 7]. Several prophylactic COVID‐19 vaccines were rapidly developed due to the global impact of the COVID‐19 pandemic [8].

With emerging variants showing increased transmission and disease severity, hopes around the world rest on higher vaccination rates to reduce disabilities and mortality from the COVID‐19, as well as reduce the prevalence of long‐term symptoms of COVID‐19 [9]. Several preventive COVID‐19 vaccines have been developed using distinct technology platforms, each with unique immunogenicity profiles and potential side effects. These vaccines include mRNA–based vaccines (e.g., Pfizer‐BioNTech BNT162b2 and Moderna mRNA‐1273), which provide genetic instructions for the synthesis of the spike protein [10, 11]. Viral vector vaccines (e.g., Oxford‐AstraZeneca ChAdOx1 nCoV‐19 and Janssen Ad26.COV2), which use modified adenoviruses to deliver spike protein genes [12, 13]. Inactivated virus vaccines (e.g., Sinopharm BBIBP‐CorV and Sinovac CoronaVac), which contain chemically inactivated particles of SARS‐CoV‐2 [14, 15]. Protein subunit vaccines (e.g., Medigen MVC‐COV1901), which deliver purified viral proteins to induce immune responses [15, 16]. Each platform has distinct immunogenicity profiles and potential adverse event patterns.

Although vaccine research has progressed rapidly, public acceptance and negative attitudes toward COVID‐19 vaccines remain significant challenges. Willingness to receive the COVID‐19 vaccine is recognized as a key issue in predicting the success of the vaccination program, and this acceptance depends on several factors such as previous vaccination experiences, education and knowledge, and risk perception [17, 18].

Anti‐SARS‐CoV‐2 antibodies decrease after the second dose of COVID‐19 vaccination, mainly as a function of time and antibody class [17, 19]. This point to the need for more efficient vaccination strategies to provide a booster dose in reducing the effects of weaning immunosuppression [20, 21].

Although the efficacy of these vaccines has been proven, monitoring for side effects is important for both clinics and the public; better control of the dissemination vaccine information through social networks helps reduce mistrust [22]. According to reports, these side effects are usually mild and temporary, including pain, fatigue, swelling and redness at the injection site, and headache. Despite these common side effects, various case reports and cross‐sectional studies have reported the most serious side effect of these vaccines [23–25]. Vaccine‐associated ocular disease is a rare known adverse event that has previously been described following COVID‐19 vaccination [26–32].

We now consider the findings of Bolletta et al. [33], who reported 34 cases of ocular complications—including uveitis, multiple evanescent white dot syndrome (MEWDS), acute macular neuroretinopathy (AMN), and retinal vein occlusion—after various COVID‐19 vaccines. This review includes reported ocular adverse events in all four categories, allowing for a comparative analysis of vaccine‐specific risks. Their study strengthens the evidence base and complements our synthesis by confirming similar patterns of post vaccination ocular inflammation across vaccine types. This systematic review seeks to comprehensively summarize the spectrum of ocular complications reported in humans after COVID‐19 vaccination.

## 2. Materials and Methods

### 2.1. Study Design and Search Strategy

The aims of this systematic review was to describe ocular occurrence after COVID‐19 vaccines and to investigate the role of treatments and demographic factors. Eligibility criteria were all human case reports of subjects receiving the COVID‐19 vaccine (all types, duration, and doses) and showing visual manifestations. Clinical manifestation were classified depending on the detected disease, the interval from vaccination to onset of symptoms, treatments, and outcome. This systematic review search strategy was registered in the PROSPERO database (Supporing Information 1: Appendix 1).

### 2.2. Data Sources

Two researchers performed a comprehensive search on the articles published until 29.01.2023 in the PubMed, Science Direct, and Web of Science (WOS) databases. The search strategy is described in Table 1.

**Table 1:** Search strategy.

Number	Date	Databases	Search query		Number of results
1	29.01.2023	**PubMed**	#1	“Visual OR visual loss OR blindness OR Amaurosis OR Usher Syndrome OR Wolfram Syndrome OR Hemianopsia”	—
			#2	“COVID‐19 Vaccine” OR “SARS‐CoV Vaccine ^∗^”	—
			#3	#1 AND #2	**5973**

2	29.01.2023	**Science Direct**	#1	“Visual OR Visual Loss OR Blindness OR Amaurosis OR Usher Syndrome OR Wolfram Syndrome OR Hemianopsia”	—
			#2	“COVID‐19 Vaccine” OR “SARS‐CoV Vaccine ^∗^”	—
			#3	#1 AND #2	**1214**

3	29.01.2023	**WOS**	—	“Visual OR Visual Loss OR Blindness OR Amaurosis OR Usher Syndrome OR Wolfram Syndrome OR Hemianopsia” AND “COVID‐19 Vaccine” OR “SARS‐CoV Vaccine ^∗^”	**3055**

*Note:* The bold is used for emphasis. The symbol “ ^∗^” is used to expand the search by finding words that start with the same letters.

### 2.3. Study Selection

Two researchers independently removed duplicates by manual screening. We excluded articles that were not written in English. Studies in which vision problems were secondary to other systemic conditions such as tumors were also excluded. Titles and abstracts were screened, and the full text of relevant articles was obtained. Abstracts that were not published as full manuscripts and review articles were excluded. No gray sources (e.g., conference abstracts, theses, or preprints) were included in this review. Titles and abstracts were independently screened by reviewers and the full text of relevant articles was obtained. The abstracts not published as full manuscripts, or reviews were excluded.

### 2.4. Data Extraction

Two investigators individually inspected full texts of eligible articles. The following data were collected from full text articles: (i) cases age and sex and disease history, (ii) COVID‐19 vaccine (type, dose, and the time of injection), (iii) diagnosed vision problems, (iv) treatment, and (v) patients following up outcomes.

### 2.5. Quality and Risk of Bias Assessment

Risk of bias in studies was assessed using “JBI Critical Appraisal Tools” and paper with high bias were omitted (Supporting Information 2: Appendix 2).

### 2.6. Results of Search

**Figure 1:**
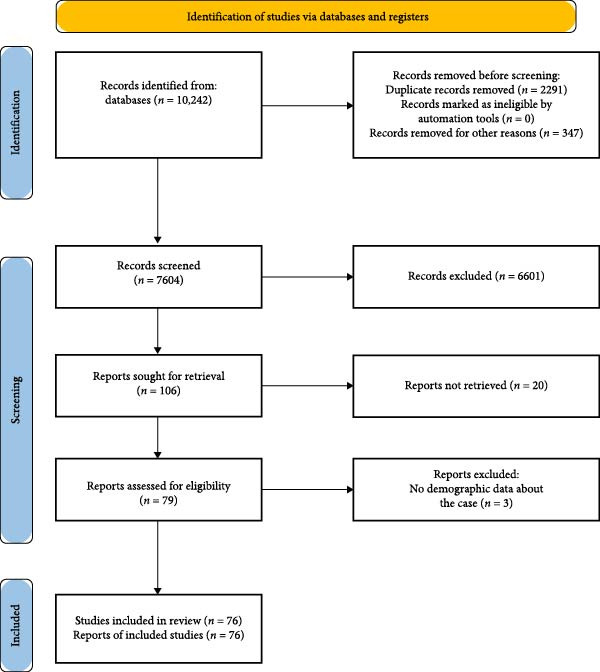
Flow diagram for this systematic reviews which included searches of databases and registers only.

Initially, 10,242 articles were identified. After removing duplicates (2291 articles), papers that met all inclusion criteria were selected; finally, 76 articles were eligible (73 case reports and three case series that among them 107 cases were reported; Figure 1, Table 2). Table 3 facilitates comparison of vaccine‐associated ocular adverse events and highlights patterns observed in the literature.

**Table 2:** Data extracted from case studies with ocular complication following COVID‐19 vaccination.

Author	Age (years)	Sex	Vaccine type and the time of complications occurrence	Other diseases	Detected disease (involved eye)	Treatment	Follow‐up	Assessed blood factors
Abdin et al. [34]	76	Female	48 h after the first dose of AstraZeneca vaccine	Hypothyroidism	CRAO (L)	Pentoxifylline (PTXF), Ophthalmic dorzolamide, Aspirine	No data	Unremarkable
Abousy et al.[26]	73	Female	2 weeks after the second dose of Pfizer vaccine	Bilateral corneal endothelial graft	DMEK (B)	Prednisone Acetate Ophthalmic, Muro 128 5% Ointment	No data	No data
Alkwikbi et al. [35]	18	Female	1 week after the second dose of Pfizer vaccine	No data	HSV keratitis (R)	No data	No data	No data
Alkwikbi et al. [35]	40	Male	1 week after the second dose of Pfizer vaccine	Allergy	Dendritic epithelial keratitis (R)	No data	Improvement after 3 weeks	No data
Alkwikbi et al.[35]	32	Male	1 week after the second dose of AstraZeneca vaccine	Healthy	HSV keratouveitis (R)	Prednisone, Cyclopentolate	No data	No data
Alkwikbi et al. [35]	29	Female	1 week after the second dose of Pfizer vaccine	No data	HSV keratitis (L)	Ganciclovir ophthalmic gel, Acyclovir Oral	Improvement	No data
Bellur et al. [27]	64	Female	Rapidly after the first dose of Moderna vaccine	No data	AMN (B)	Prednisone	Mild improvement	No data
Bouhout et al. [36]	28	Female	2 weeks after the second dose of Moderna vaccine	HSV	MEWDS (R)	No treatment	Improvement after 1 month	No data
Bouhout et al. [36]	27	Male	22 days after the first dose of Pfizer vaccine	Healthy	MEWDS (R)	No treatment	Improvement after 3 months	No data
Bouhout et al. [36]	26	Female	3 days after the second dose of Pfizer vaccine	No data	Increased blind spot and Photopsia	No data	Improvement after 4 months	No data
Chen et al. [28]	48	Female	1 month after the first dose of AstraZeneca vaccine	Healthy	Usac’s syndrome (B)	Brimonidine tartrate	Improvement after 3 months	No data
Cunha et al. [37]	50	Male	2 weeks after the second dose of Pfizer vaccine	Healthy	TVL (B)	Verapamil	Fully improvement after 2 days	Unremarkable
Druke et al. [38]	23	Female	3 days after the second dose of the Moderna vaccine	No data	AMN (B)	Difluprednate ophthalmic, Prednisone	Improvement after 2 weeks	No data
Elhusseiny et al. [29]	51	Female	1 day after the second dose of Pfizer vaccine	Hypertension	NA‐ION with macular star (L)	Prednisone	Improvement after 8 weeks	Unremarkable
Elhusseiny et al. [29]	18	Female	5 days after the second dose of the Sinopharm vaccine	Oligoarticular juvenile idiopathic arthritis	Acute uveitis (B)	Steroid treatment	Improvement	Unremarkable
Endo et al. [30]	52	Male	15 days after the first dose of Pfizer vaccine	Healthy	CRVO (L)	Bevacizumab, Oral Apixaban	Improvement	Unremarkable
Fard et al. [39]	67	Female	Rapidly after the second dose of Moderna vaccine	Hypothyroidism, Hodgkin’s lymphoma, HZO, Guillain–Barré syndrome	Visual decline and patchy stromal	Verapamil, Fluorometholone, *Mycobacterium Chelonae*, Amikacin eye drops, Azithromycin	Improvement	Unremarkable
Fard et al. [39]	52	Male	24–48 h after the second dose of BNT162b2 vaccine	Herpetic keratitis	HSV keratitis (R)	Acyclovir, Trifluridine, Prednisone Acetate Ophthalmic, Fluorometholone, Valaciclovir	Improvement	No data
Forshaw et al. [40]	94	Female	2 weeks after Pfizer vaccine	DMEK (B)	DMEK rejection	Tobramycin–Dexamethasone Ophthalmic, Ketorolac	Poor response resulted in re‐DMEK transplantation, starting in L eye	No data
Gabrielle et al. [41]	25	Female	24 h after the first dose of AstraZeneca vaccine	Healthy	AMN (B)	No data	No data	Unremarkable
Goyal et al. [42]	34	Male	1 week after the second dose of AstraZeneca vaccine	No data	MFC (B)	Corticosteroid	Improvement after 2 weeks	Unremarkable
Grunenwald et al. [43]	65	Female	2 days after the first dose of BNT162b2 vaccine, and after the second dose	Resection of right lobe of the thyroid due to toxic adenoma, seropositive rheumatoid arthritis, Amnesic stroke	Orbital inflammatory disease and ocular hypertension (B)	Brinzolamide/Timolol, MethylPrednisone	Improvement after 6 months	Unremarkable
Hasegawa et al. [44]	54	Female	1 day after the second dose of Pfizer vaccine	Healthy	UAIM (R)	Betamethasone	Improvement after 3 months	No data
Hwang [45]	21	Male	22.8 days after (5 s 1 first) Pfizer vaccine	Healthy	Uveitis	Dexamethasone, Prednisone	Improvement after 7 days	Unremarkable
Inagawa et al. [46]	30	Female	13 days after the first dose of Pfizer vaccine and 3 days after the second dose	No data	MEWDS (L)	Fluorometholone Ophthalmic, Betamethasone sodium phosphate/Fradiomycin Sulfate	Improvement after 2 months	Unremarkable
Iwai et al. [47]	78	Male	2 days after the first dose of Pfizer vaccine	Healthy	ARN (R)	Antiviral agents and corticosteroids	Need repeated vitrectomy using silicone oil which occurred after the second vaccine	No data
Jalink et al. [48]	53	Male	1 day after the booster shot of Moderna vaccine	No data	Orbital inflammation and dacryoadenitis (R)	Amoxicillin/clavulanic‐acid/Prednisone	Improvement after 2 weeks	C‐reactive protein (CRP) was slightly elevated
Jumroendararasame et al. [49]	42	Male	1 h after the second dose of Sinovac vaccine	Dyslipidemia	TVL (B)	No data	Improvement after 2 h	No data
Kang et al. [50]	64	Male	3 days after the first dose of Pfizer vaccine	Diabetes and hypertension	BRAO (R)	Aspirin, Atorvastatin Brimonidine Tartrate, Dorzolamide hydrochloride, and timolol	Improvement after 3 months	No data
Khochtali et al. [51]	24	Female	5 days after the first dose of	No data	BRAO, acute foveolitis (L)	Prednisone	Improvement	No data
Kim et al. [52]	21	Male	2 days after the first dose of Pfizer vaccine	Healthy	Cataract (B)	Intravenous immunoglobulin (IVIG), Steroids, and Operation	Improvement after 1 month post operation	CRP, erythrocyte sedimentation rate, lactate dehydrogenase, and lymphopenia were elevated
Leber et al. [53]	—	—	Sinovac vaccine	No data	Acute thyroiditis and optic neuritis (B)	Corticosteroids	No data	No data
Lee et al. [54]	83	Female	2 days after the second dose of Pfizer vaccine	Hypertension	Neuroretinitis (B)	MethylPrednisone, Prednisone	Improvement after 6 months	Unremarkable
Lee et al. [54]	55	Female	6 days after the first dose of AstraZeneca	Cataract surgery (B)	Transient corneal edema (B)	Prednisone	Improvement	No data
Lee et al. [54]	34	Female	12 days after the first dose of Pfizer	No data	CRAO and CRVO (L)	MethylPrednisone, Trental, and Eliquis	Improvement after 3 weeks	Erythrocyte sedimentation rate (ESR) was slightly elevated
Lin et al. [55]	36	Female	2 days after the first dose of Madigan Vaccine Biologics Corporation (MVC) vaccine	No data	MEWDS (R)	No treatment	Improvement after 1 month	No data
Lin et al. [55]	61	Female	7 days after the first dose of AstraZeneca	Hypertension, cataract surgery (B)	NA‐AION	Prednisone	Improvement after 6 weeks	Unremarkable
Liu et al. [56]	49	Female	2 weeks after the first dose of ChAdOx1 vaccine	Healthy	Optic neuritis (B)	Prednisone	Fully improvement after 7	Unremarkable
Lo et al. [57]	42	Male	12 days after the first dose of Pfizer vaccine	Healthy	ARN (L)	Intravitreous foscarnet, Valacyclovir, Prednisone, Dexamethasone, Atropine/diphenoxylate, and barrier laser for retinal tear	Improvement	No data
Maleki et al. [58]	79	Female	2 days after the second dose of Pfizer vaccine	Osteoarthritis + osteoporosis	NA‐AION	Steroid, Prednisone	Improvement after 3 months	ESR and CRP were elevated
Maleki et al. [58]	33	Female	10 days after the second dose of Moderna vaccine	Cataract	Acute zonal occult outer retinopathy (AZOOR)	Dexamethasone	Improvement	ESR and CRP were elevated
Mambretti et al. [59]	22	Female	2 days after receiving the first dose of Vaxzevria	Healthy	AMN (R)	No data	No data	No data
Mambretti et al. [59]	28	Female	2 days after receiving the first dose of Vaxzevria	Healthy	AMN (R)	No data	No data	Unremarkable
Mohammadzadeh et al. [60]	36	Female	7 days after the first dose of Sinopharm vaccine	PKP (L)	Graft rejection (L)	Betamethasone Topical, Acyclovir Oral	Improvement after 14 days	No data
Mohammadzadeh et al. [60]	54	Female	7 days after the first dose of Sinopharm	PKP secondary to HSK	Graft rejection	Betamethasone, Acyclovir Oral	Fully improvement after 2 weeks	No data
Nagaratnam et al. [61]	36	Female	14 days after the first dose of AstraZeneca	Healthy	ADEM with bilateral optic neuritis (B)	Prednisone	Improvement after the 3 months	No data
Nahata et al. [62]	28	Female	2 weeks after the first dose of AstraZeneca	Eye laser	Transplant rejection (L)	Prednisone, Homatropine ophthalmic	Improvement after the 5 weeks	No data
Ninet et al. [63]	30	Female	1 months after the second dose of BNT162b2 vaccine	Ectopia Lentis, transscleral fixation	MEWDS (R)	No data	Improvement after 2 months	No data
Pan et al. [64]	50	Female	5 days after the first dose of Sinopharm Vaccine	Healthy	Uveitis (B)	Triamcinolone acetonide injection, Prednisone	Improvement after 5 weeks	Unremarkable
Pur et al. [65]	34	Male	2 days after the first dose of Pfizer vaccine	LASIK	BRVO (R)	No data	Improvement after 4, 7 and 10 months	Unremarkable
Rallis et al. [66]	68	Female	3 days after the first dose of BNT162b2 vaccine	DSEK	Graft rejection (L)	Dexamethasone and Acyclovir Oral	Improvement after 3‐weeks	No data
Renisi et al. [67]	23	Male	5 h after the first dose of BNT162b2 vaccine, 14 days after the second BNT162b2 dose	Panic attacks treated with benzodiazepines	Periocular dermatitis (L) and anterior uveitis	Glucocorticoids, Dexamethasone eye drops, Cycloplegic Agent, Glucocorticoid	Fully improvement after 6 weeks	Unremarkable
Rennie et al. [68]	21	Female	3 days after receiving the second dose of the Moderna COVID‐19	Healthy	AMN (B)	Difluprednate Ophthalmic and Brimonidine	No data	No data
Reshef et al. [69]	68	Male	4 days after the second dose of BNT162b2 vaccine	Healthy	Periorbital edema and erythema (L)	Prednisone	No data	Unremarkable
Reshef et al. [69]	33	Female	1 day after the second dose of Moderna vaccine	Orbital inflammation following Influenza vaccine	Inflammation and enlargement of the left medial and lateral rectus, and superior oblique muscles (L)	Prednisone	No data	Unremarkable
Reshef et al. [69]	13	Male	1 day after the first dose of BNT162b2 vaccine	Idiopathic orbital inflammation	Orbital inflammation with left lacrimal gland enlargement and intraconal fat stranding (L)	Prednisone	No data	Unremarkable
Richardson‐May et al. [70]	82	Male	1 day after after his first dose of AstraZeneca vaccine	HSK	HSV keratitis	Ganciclovir ophthalmic gel	Improvement	No data
Ryu and Kim [71]	87	Male	2 days after the second dose of BNT162b2 vaccine	Varicella zoster virus keratitis	HZO	Valacyclovir Oral, Corticosteroids	Improvement after 2 weeks	No data
Ryu and Kim [71]	70	Female	A few hours after the first dose of AstraZeneca	Cataract surgery	Varicella zoster virus‐associated anterior uveitis (B)	Intravitreal Ranibizumab injection	Improvement after 3 months	Unremarkable
Sacconi et al. [72]	74	Female	48 h after the second dose of Moderna vaccine	Healthy	CRVO (R)	Intravitreal Ranibizumab injection	Improvement after 5 weeks	No data
Salai et al. [73]	48	Female	127 days after the first dose of BNT162b2	Palpitations	NA‐AION	No data	No data	No data
Sanjay et al. [74]	40	Male	2 days after the first and 3 days after the second dose of AstraZeneca vaccine	CSCR and laser	CSCR (B)	Eplerenone	Improvement after 2 months	Unremarkable
Sanjay et al. [74]	50 s	Female	Fast after the first dose of AstraZeneca vaccine	NA‐AION	NA‐AION (L)	Aspirin, Prednisone	Improvement after 1 months	Unremarkable
Santovito and Pinna [75]	No data	Male	3 days after the second dose of Pfizer vaccine	No data	TVL (B)	No treatment	Improvement after a few hours	No data
Sarigul Sezenoz et al. [76]	38	Female	Second dose of Pfizer(R)‐BioNTech COVID‐19 vaccine	Healthy	TVL (L)	No Data	Improvement after 1 week	No data
Savino et al. [77]	64	Female	5 days after the second dose of Pfizer (R)‐BioNTech COVID‐19 vaccine	No data	Scleritis (R)	Prednisone	Fully improvement after 6 months	Unremarkable
Savino et al. [77]	58	Female	The second dose of mRNA BNT162b2 COVID‐19	Latex allergy, Hashimoto thyroiditis	Scleritis (B)	Ophthalmic phenylephrine, Dexamethasone	Improvement after 8 days after	Unremarkable
Savino et al. [77]	45	Female	Days after the first dose of mRNA COVID‐19 vaccine	Eye proptosis (L)	Myositis (L)	Oral nonsteroidal anti‐inflammatory drugs (NSAIDs)	Improvement after 3 months	Unremarkable
Sarigul Sezenoz et al. [76]	38	Female	After the second dose of Pfizer vaccine	No data	A temporal hemifield defect (L), TVL (R)	No treatment	Improvement after 1 week	No data
Shah et al. [78]	27	Female	A few days after the first dose of Pfizer vaccine and after the second one	No data	CRVO (B)	Ranibizumab injection	Improvement	No data
Shah et al. [78]	27	Female	A few days after the first dose of Pfizer vaccine	Healthy	CRVO	Ranibizumab injection	Improvement	No data
Shan et al. [79]	19	Male	3 weeks after the second dose of Sinovac vaccine, 7 days after the third dose	Rubella virus, HSV	Keratitis (B)	Ganciclovir, Cyclosporine, and Acyclovir	Improvement after 1 week	Unremarkable
Smith et al. [80]	30	Female	1 months after the second dose of Pfizer vaccine	Ectopia lentis, transscleral fixation (L)	MEWDS (R)	Corticosteroids	Improvement after 2 months	No data
Sodhi et al. [81]	43	Male	3 days after the first dose of AstraZeneca	Healthy	CRVO (L)	Triamcinolone Acetonide injection, Atargeted laser after 6 month	Improvement after 6 months	ESR, CRP, and d‐dimer were elevated
Sonmez et al. [82]	41	Female	3 days after the first dose of AstraZeneca vaccine	Healthy	CRVO (R)	Aspirin, Heparin	Improvement after 1 month	No data
Subramony et al. [83]	22	Female	3 days after the first dose of Moderna vaccine	Myopia	RRD (B)	Bilateral vitrectomies with a transconjunctival pars plana approach	Improvement after 13 days	No data
Sugihara et al. [84]	38	Male	2 days after the second dose of Pfizer vaccine	Healthy	BRVO (L)	Anti‐vascular endothelial growth factor (VEGF) injection (Aflibercept)	Improvement after 13 days	Unremarkable
Subramony et al. [83]	22	Female	10 days after the secend dose of Moderna vaccine	Myopia	Retinal detachments (B)	Bilateral vitrectomies with a transconjunctival pars plana approach	Improvement after 13 days	No data
Sung et al. [85]	25	Female	10 days after the third dose of Pfizer vaccine	Healthy	CRVO (L)	Anti‐VEGF injection	Mild improvement after 6 months	Unremarkable
Tanka et al. [86]	71	Female	1 day after the second dose of Pfizer	BRVO and ME (L)	BRVO(L),	One‐time dose of IVA	Improvement	No data
Tanka et al. [86]	72	Male	1 day after first dose of	BRVO without ME	ME and BCVA (R)	Ranibizumab injection	Improvement	No data
Tomishige et al. [87]	38	Female	3 weeks after the first dose of Sinovac	No data	MEWDS (R)	Prednisone	Improvement after 4 weeks	No data
Tsukii et al. [88]	55	Female	7 days after the first dose of Pfizer vaccine	Healthy	NA‐AION (R)	No treatment	Improvement after 2 months	Unremarkable
Valenzuela et al. [89]	20	Female	2 days after the second dose of Pfizer vaccine	Healthy	AMN (B)	Prednisone and Diphenhydramine	Improvement after 2 weeks	No data
Valsero Franco and Fonollosa [90]	53	Male	12 weeks after the first dose of Pfizer vaccine	Healthy	NA‐AION	Acetazolamide	Improvement after 3 months	Unremarkable
Valsero Franco and Fonollosa [90]	65	Male	12 days after first vaccination	No data	NA‐AION	No treatment	Improvement after 1 month	Unremarkable
Vinzamuri et al. [91]	35	Male	After both dose of AstraZeneca vaccine	No data	PAMM (B)	No Data	Improvement after 3 weeks	Unremarkable
Wang et al. [92]	21	Female	6 weeks after the first dose and 3 weeks after the second Sinopharm vaccine	Healthy	Optic neuritis (R)	Prednisone	Fully improvement after 1 month	Unremarkable
Wang et al. [92]	39	Female	3 weeks after the first dose	Healthy	Optic neuritis (R)	Prednisone	Fully improvement after 1 month	Unremarkable
Wasser et al. [93]	73	Male	13 days after the first dose of BNT162b2 vaccine	PKP	PKP (L)	Dexamethasone and Prednisone	Improvement after 1 week	No data
Wasser et al. [93]	56	Male	14 days after the first dose of BNT162b2 vaccine	PKP (B)	PKP (R)	Dexamethasoneand Prednisone	Fully improvement after 1 month	No data
Yamaguchi et al. [94]	30	Female	2 weeks after the second dose of Pfizer vaccine	No data	VKHD	Steroid therapy	Fully improvement after 3 months	No data
Yasuda et al. [95]	67	Female	1 day after second dose of Pfizer vaccine	Healthy	MEWDS	No treatment	Improvement after 2 weeks	No data
Yucel Gencoglu and Mangan [96]	40	Female	1 week after the first dose of Pfizer vaccine	Healthy	Orbital pseudotumor	Corticosteroid	Improvement after 1 month	Unremarkable
Zheng et al. [97]	62	Male	7 days after the Pfizer vaccine	No data	ARN	No data	No data	No data

*Note:* ME, secondary macular edema; RRD, macula‐Off rhegmatogenous retinal detachment.

Abbreviations: ADEM, acute disseminated encephalomyelitis; AMN, acute macular neuroretinopathy; ARN, acute retinal necrosis; BRAO, branch retinal artery occlusion; CRAO, central retinal artery occlusion; CRVO, central retinal vein occlusion; CSCR, central serous chorioretinopathy; DMEK, Descemet’s membrane endothelial keratoplasty; DSEK, Descemet stripping endothelial keratoplasty; HSV, herpes simplex virus; HZO, herpes zoster ophthalmicus; MEWDS, multiple evanescent white dot syndrome; MFC, multifocal choroiditis; NA‐ION, nonarteritic anterior ischemic optic neuropathy; PKP, penetrating keratoplasty; TVL, transient visual loss; UAIM, unilateral acute idiopathic maculopathy; VKHD, Vogt–Koyanagi–Harada disease.

**Table 3:** Summary of reported ocular complications following COVID‐19 vaccination across included studies.

Ocular complication	Number of studies reporting	Associated vaccines	Typical onset (days)	Notes
Uveitis	18	Pfizer‐BioNTech, Moderna, AstraZeneca	3–14	Most common complication; includes various subtypes
MEWDS	7	Pfizer‐BioNTech, Moderna	5–10	Often self‐limiting
AMN	6	Janssen, Moderna	2–7	Predominantly in young females
Retinal vein occlusion	5	AstraZeneca, Moderna	7–12	Includes BRVO and CRVO
Optic neuritis	4	Pfizer‐BioNTech, AstraZeneca	6–14	Variable visual recovery
VKH syndrome	3	Pfizer‐BioNTech	10–14	Reactivation and new‐onset cases
Other (e.g., episcleritis, scleritis)	8	Various	4–15	Mixed presentations

*Note:* The table describes the types of complications, the number of studies that have reported each condition, the types of vaccines involved, the usual starting intervals, and the relevant clinical points.

Abbreviations: AMN, acute macular neuroretinopathy; BRVO, branch retinal vein occlusion; CRVO, central retinal vein occlusion; MEWDS, multiple evanescent white dot syndrome; VKH, Vogt–Koyanagi–Harada syndrome.

### 2.7. Ocular Complications After COVID‐19 Vaccination

The majority of the cases were people who had injected the Pfizer‐BioNTech COVID‐19 vaccine (Pfizer) vaccine (57%); at the second level was the Oxford/AstraZeneca, ChAdOx1‐S, (AstraZeneca; 19.63%); the third, Moderna Covid19 Vaccine (Moderna; 12.15%). By examining the studies, we found that the percentage of women was more than men (63.80%) and the average age was (44.88 ± 19.21) which was calculated separately in two genders, women (43.50 ± 18.91) and men (47.14 ± 20.03). Among these patients, Lebers’ patient had a history of hospitalization due to severe COVID‐19 [53]. In the following, we will examine the reports of each type of vaccine separately (Table 2).

### 2.8. Pfizer Vaccine

Reviewing the related studies showed that 61 patients (44 studies) had ocular complications following Pfizer vaccination. The mean age of patients was 47.55 ± 20.46 and 52.46% of them were women. A total of 19 patients showed ocular complications after the first dose of vaccine, 34 patients after the second dose, one patient after the third one [85], and five patient after both doses [43, 45, 46, 67, 79]. In a study by Zheng et al. [97], it was noted that acute retinal necrosis (ARN) symptoms started 7 days after receiving the COVID‐19 vaccine, and also in a study by Forshaw [40], DMEK rejection 2 weeks after Pfizer vaccine was pointed out. The onset of symptoms after the first injection in 47.37% of cases started in less than 1 week [47, 50, 52, 65, 66, 77, 78, 88, 96, 98], one to 2 weeks (26.31%) [57, 90, 93, 99], more than 2 weeks (15.79%) [30, 100], and less than 24 h (10.53%) had the next ranks, respectively.

Second dose induced ocular problems began immediately (in first 24 h) in eight patients [29, 44, 67, 86, 95, 101], in less than a week for six patients [35, 39, 54, 58, 69, 75, 77, 84, 89, 100], one to 2 weeks for five [26, 37, 90, 98, 100], and in less than a month for four [63, 80, 100]. In three studies, the exact time after the second dose was not reported [81, 83, 84].

According to Shah et al.’s [78] case report, 13 days after the first dose of Pfizer vaccine and 3 days after the second dose a patient referred to an eye clinic due to blurred vision. Evaluation of the underlying disease presented six hypertensive cases [29, 43, 54, 98, 100] and 10 patients with a history of eye diseases [26, 39, 40, 63, 65, 66, 69, 77, 80, 93].

The clinical examination of the patients showed that the injection of Pfizer vaccine causes ocular complications; the most frequent eye problems are as follows: 10 cases of branch retinal artery occlusion (BRAO) [50, 65, 84, 86, 98, 100], five cases of MEWDS [36, 46, 63, 80, 95], keratitis in five cases [35, 39, 79], transient visual loss (TVL) and graft rejection in seven [37, 75, 76, 101] and five [26, 40, 66, 93] studies, respectively.

Treatment of patients with routine drugs showed that almost all patients recovered within a reasonable time frame, studies even had reported a case that recovered without any treatment [75].

### 2.9. AstraZeneca Vaccine

A total of 21 patients with a mean age of 46.05 ± 17.61 years had shown ocular complications. A total of 17 patients showed ocular complications after the first dose of Pfizer vaccine, second dose induced multifocal choroiditis (MFC) 1 week [42] and herpetic keratouveitis after the vaccination [35], and two patients showed indications after both doses [74, 91]. The onset of symptoms after the first injection happened in less than 24 h [41, 71, 102], in less than 1 week [34, 70, 81, 82, 98, 99, 103], in less than 2 weeks [28, 54, 56, 61, 62], and in about 1 month [28]. Evaluation of the underlying disease presented seven eye diseases history [62, 70, 71, 74, 99, 102, 103].

Clinical examination of patients showed that AstraZeneca vaccination caused ocular complications such as AMN [41, 60], nonarteritic anterior ischemic optic neuropathy (NA‐ION) [98, 102, 103], and optic neuritis [54, 56, 61]. Follow‐up in many studies showed that most symptoms improved due to the treatment. In a case report, Sanjay et al. [102] presented a 40‐year‐old male with a history of central serous chorioretinopathy (CSCR) who suffered from blurring of vision 2 days after AstraZenica injection. Six weeks later, he took the second dose of vaccine, and blurring of vision developed 3 days later [74]. In another study, following the first dose, the patient noticed a mild blurring of vision that after 4 weeks and receiving the second dose, the visual symptoms progressed further, clinical examination had detected unilateral acute idiopathic maculopathy (UAIM) and AMN [91]. Recovery of this group patients requires at least a 2‐week period. Intrestingly, high presentage of this group patients had reported previous occular complications.

### 2.10. Moderna Vaccine

A total of 13 case with the mean age of 38.31 ± 19.13 years reported ocular complications after the first (rapidly and 3 days) dose [27, 83] and the second doses of Moderna vaccine. Symptoms occurred after the second injection in three periods, less than 24 h [39, 48, 69], about 1 week [38, 41, 68, 72, 83], and in one to 2 weeks [36, 58, 101]. A 84.61% of patients were women and eye disease was observed as the most underlying disease in four patients [58, 69, 83].

Clinical diagnose showed that AMN [27, 38, 68] and macula‐off rhegmatogenous retinal detachment (RRD) [83] were the most common diseases. Follow‐up showed that the treatments had a partial effect after at least 2 weeks.

### 2.11. Sinopharm Vaccine

Reviewing the results demonstrated that eye problems appeared in nine cases after Sinopharm vaccine injection [49, 53, 60, 64, 87, 92, 104]. The mean age of these cases was 39.43 ± 11.59 years and 77.78% of them were woman. Eye complications were observed after the injection of first [60, 64, 87, 92], second [49, 104], and even both doses [92]. The most common eye diseases in this group were acute uveitis [64, 104], optic neuritis [92], and graft rejections [60].

### 2.12. Other Vaccines

There are other case reports that had investigated the role of other vaccines in eye diseases. In a study by Mambretti et al. [59] AMN in two young women, 2 days after receiving Vaxzevria COVID‐19 vaccination, was discussed. In another study, MEWDS following Medigen vaccine was detected.

In a multinational case series of 70 patients, within 14 days Following COVID‐19 vaccination, the mean age was 51 years and the most common problem were anterior uveitis (58.6%), uveitis (12.9%), and Scleritis (10.0). A 54.1% had a previous history of ocular inflammatory event and treatments were not effective in 92.9%, whereas 6.3% had reported visual acuity reduction. Pfizer, AstraZeneca, Moderna, Sinopharm, and Covaxin accounted for 40%, 17%, 10%, 2%, and 1%, respectively [105].

## 3. Discussion

Since the initial outbreak in 2019, the virus has affected millions of adults, so the world faced severe challenges in a short time. Meanwhile, with the development of preventive vaccines, complications were observed, some of which were significant [106–108]. Reviewing the included articles showed that ocular problem is one of the serious side effect of vaccines injection. Although several systematic reviews have examined ocular complications after COVID‐19 vaccination, the present study provides a comprehensive and unique synthesis. Previous reviews, such as those by Haseeb et al. [31] and Lamptey [106], have identified similar complications but were limited in scope or vaccine diversity. In contrast, the present study provides a more comprehensive synthesis, including 107 individual cases from 76 studies across four vaccine platforms (mRNA, viral vector, inactivated, and protein subunit). By stratifying findings by demographic factors, vaccine type, dose, and ocular diagnosis, the review reveals subtle patterns. In this study, sensitivity was increased in women and older adults, and comparative insights into vaccine‐specific adverse events were provided. The inclusion of a registered protocol in PROSPERO, detailed assessments of risk of bias, and visual mapping further enhance the methodological rigor and transparency of this work, distinguishing it from previous reviews.

According to Table 2, the largest number of reports was related to Pfizer vaccine and the next rank belonged to AstraZeneca. This trend is consistent with findings from Haseeb et al. [31], who also reported a predominance of ocular complications linked to mRNA and viral vector vaccines. However, Moll et al. [109] found AstraZeneca and Sputnik V to be more frequently associated with adverse events in a Mexican cohort, highlighting regional variation in vaccine distribution and reporting. In this context, different studies had different opinions. For example, in a study on 4024 Mexicans, the most identified complications were related to the AstraZeneca vaccine (85%), followed by Sputnik V (Gam‐COVID‐19‐Vac) (80%), while at Dose 2, Maderna was the vaccine with the highest rate of side effects (88%) [109]. In another study by Medeiros et al. [110], they had concluded that mRNA–based vaccines were linked with localized adverse effects such as injection site discomfort, while viral vector‐based vaccines were more prone to imparting systemic side effects such as headache and fatigue. More specific investigation in different studies showed that the most ocular side effects were seen after Pfizer, AstraZeneca, or Moderna vaccines [31], which are consistent with our study.

The study by Bolletta et al. [33] represents one of the first and most comprehensive attempts to document ocular inflammatory events following COVID‐19 vaccination. Their findings closely mirror ours in terms of complication types and timing, reinforcing the consistency of postvaccination ocular inflammation across different populations and vaccine platforms. Their observational analysis of 34 cases revealed a diverse range of complications. This study showed complications such as anterior uveitis, MEWDS, AMN, and retinal vein occlusion with a median onset of symptoms approximately 9.4 days after vaccination. Importantly, while most cases were associated with the Pfizer‐BioNTech vaccine, similar adverse events were reported after the AstraZeneca, Moderna, and Janssen vaccines, highlighting the need for cross‐platform safety assessments. Bollettaet al. [33] also cited cases of new‐onset and reactivated eye diseases, such as Behçet’s disease and VKH syndrome, suggesting that COVID‐19 vaccines may induce immune responses in susceptible individuals. Their findings reinforce the importance of ongoing surveillance and provide valuable clinical insight into our understanding of vaccine‐associated ocular inflammation.

In order to investigate the cause of more eye complications in some vaccines, it is important to pay attention to the type of ocular diseases and the more effective dose. Retinal vascular occlusion was the most ocular side effect [30, 34, 50, 65, 72, 81, 82, 84–86, 98, 100, 111]. These studies, like ours, suggest a possible link between vaccine‐induced inflammation and thrombotic events. Mechanistic hypotheses proposed by Chen et al. [28] and Thakar and Bhattacharya [112] support this association, pointing to platelet activation and spike protein‐mediated procoagulant responses. Studies have reported that systemic thrombotic complications are associated with COVID‐19 vaccination as a result of inflammation‐induced thrombosis [28]. Other hypotheses are the interactions between vaccine and platelets or platelet factor 4 (PF4), and the vaccine S proteins‐induced procoagulant response which themselves are known as the potent mediator of inflammation [112–114]. On this basis, inflammatory eye diseases should also have a higher rate of involvement, which studies reviewing showed high incidence of AMN [27, 38, 41, 44, 59, 68, 89, 98, 100], graft rejections [26, 40, 60, 62, 66, 93], and so on. In this regard, using corticosteroids would reduce the inflammation induced ocular diseases, which has been observed in the reviewed studies.

In evaluating the role of different doses, although the most complications were observed in the second dose, it cannot be generalized to individual vaccines. Our findings align with those of Chapin‐Bardales et al. [115] and Kelliher et al. [116], who reported increased adverse events after the second dose of mRNA vaccines. This supports the theory that immune priming from the first dose amplifies inflammatory responses upon re‐exposure [115, 116]. mRNA vaccines showed the most complications after the secondary dose, which was also reported in other articles [115–117]. This happened because the body is primed with the first dose of the vaccine, and after the second dose, the spike protein and antibodies increase the immune system’s response [118–120]. On the other hand, since antibody titers in vaccinated individual decreases overtime [22], a decrease in the incidence of ocular diseases over time is not too far off.

Examining the role of personal risk factors such as age, gender, and underlying disease showed that women and older adults are more exposed to eye problems caused by the vaccines. In this regard, in a study of 843 participants, 65% of males experienced adverse effects compared to 77% of females [121], or in a study to assess the side effects of Pfizer vaccine, it was shown that out of 1137 participants, females complained significantly more about side effects [122]. Three mechanisms underlying the higher risk of adverse events in females; the first mechanism is a higher immune response in women due to high expression of X chromosome‐linked genes, such as IFN I, innate immune responses, and T cell‐associated genes. The ACE2 and Ang‐II receptor type 2 gene are also located on the X chromosome [123, 124]. A second mechanism is the level of sex hormones, for instance testosterone can depress the innate and adaptive immune response [125, 126]. The last mechanism is the higher risk of mast cell (MC)‐associated diseases in female [127, 128].

This study did not clearly demonstrate age as a risk factor in the occurrence of eye complications, although it is interesting to note that the predominant age in the reported cases was usually greater than 25 (about 85% of cases). Some students believe that adverse reactions of the COVID‐19 vaccine were higher in older; for example, in a study following the first dose of AstraZeneca, it was showed that 50–60 years people had higher side effects than that of older or younger [128–130]. On the other hand, in a study on ocular adverse events occurred after inactivated COVID‐19 vaccines, it was shown that the mean age of patients diagnosed with ocular complications were comparable to that of other diagnosed patients [131].

Various case reports have examined the ocular complications caused by COVID‐19 vaccines, in these studies despite showing full details of the patients, the level of anti‐SARS‐CoV‐2 antibodies was not measured and tracked; perhaps measuring the titer of anti‐SARS‐CoV‐2 antibodies at the beginning of diagnosis or during treatment can show us important points about the actual function of antibodies.

Although vaccine‐induced ocular complications have been documented for various vaccines, including influenza, hepatitis B, and human papillomavirus (HPV), the spectrum and frequency of reported events following COVID‐19 vaccination appear to be more diverse and prominent [132, 133]. This may be attributed to new immunogenic platforms (such as mRNA and viral vector technologies) and the increased immune system activation they induce. Additionally, the scale of global COVID‐19 vaccination efforts and the intensity of postvaccination surveillance likely contribute to increased detection and reporting. However, most ocular complications are rare and self‐limiting. The pathophysiological mechanisms behind vaccine‐induced ocular complications are multifactorial and have not yet been fully elucidated. Several possible immunological and vascular pathways have been proposed. mRNA and viral vector vaccines stimulate strong immune responses that may inadvertently cause ocular inflammation in susceptible individuals. This involves molecular mimicry, where vaccine antigens resemble ocular self‐antigens and lead to autoimmune reactions such as uveitis or scleritis. Furthermore, spike protein‐mediated endothelial activation and platelet aggregation may contribute to thrombotic events, such as retinal vein or artery occlusion. Studies have also implicated vaccine‐induced cytokine release and MC activation in exacerbating pre‐existing eye diseases.

Although ocular complications following COVID‐19 vaccination are rare, their potential affects patient quality of life and public perception is significant. Even transient visual disturbances can cause significant anxiety and may prevent individuals from completing vaccination schedules or receiving booster doses. In the context of widespread vaccine skepticism, sporadic reports of serious eye complications can be amplified through social media and fuel misinformation. Therefore, systematic documentation and transparent reporting of such side effects is essential not only to guide clinicians in early diagnosis and management, but also to reassure the public that adverse effects are being closely monitored. By synthesizing the available evidence, this review supports informed decision‐making and helps balance the perceived risk with the proven benefits of vaccination.

In conclusion, although this article refers to the side effects caused by vaccines, we should not forget the impact vaccination on the world health. Although Pfizer and AstraZeneca had reported the most eye complications, these two vaccines had the most follow‐up and investigation among the vaccines. Inflammation and any factor affecting it important factor in the occurrence of vaccine‐induced eye disease.

## Ethics Statement

The authors have nothing to report.

## Conflicts of Interest

The authors declare no conflicts of interest.

## Funding

This article has not received any funding.

## Supporting Information

Additional supporting information can be found online in the Supporting Information section.

## Supporting information


**Supporting Information 1** The supplements are the PDF of Appendix 1, which included the PROSPERO registration file of this systematic review.

**Supporting Information 2** Appendix 2, which contain the heat map of articles’ quality assessment using JBI Critical Appraisal Tools.

## Data Availability

The datasets used and/or analyzed during the current study are available from the corresponding authors upon reasonable request.
