# Supplementary material for: A Systematic Review of Ocular Complications Following Different Types of Covid‐19 Vaccines
Source: J Immunol Res. 2025 Oct 21;2025:8766021. doi: 10.1155/jimr/8766021 (PMC12538234; doi:10.1155/jimr/8766021)
Supplement: Supplementary file 2 — Supporting Information 2 Appendix 2, which contain the heat map of articles’ quality assessment using JBI Critical Appraisal Tools. [file JIMR-2025-8766021-s002.docx]

| **Author** | **Demographic** | **History** | **Current condition** | **Diagnostic tests** | **Treatment** | **Post-intervention** | **Advers events** |
| --- | --- | --- | --- | --- | --- | --- | --- |
| Abdin et al., 2022 | Yes | Yes | Yes | Yes | Yes | NO | Not applicable |
| Abousy et al., 2021 | Yes | Yes | Yes | Yes | Yes | NO | Not applicable |
| Alkwikbi et al., 2022 | Yes | NO | Yes | Yes | NO | NO | Not applicable |
| Alkwikbi et al., 2022 | Yes | Yes | Yes | Yes | NO | Yes | Not applicable |
| Alkwikbi et al., 2022 | Yes | Yes | Yes | Yes | Yes | NO | Not applicable |
| Alkwikbi et al., 2022 | Yes | NO | Yes | Yes | Yes | Unclear | Not applicable |
| Bellur et al., 2022 | Yes | NO | Yes | Yes | Yes | Yes | Not applicable |
| Bouhout et al., 2022 | Yes | Yes | Yes | Yes | Yes | Yes | Not applicable |
| Bouhout et al., 2022 | Yes | Yes | Yes | Yes | Yes | Yes | Not applicable |
| Bouhout et al., 2022 | Yes | NO | Yes | Yes | Yes | Yes | Not applicable |
| Chen et al., 2022 | Yes | Yes | Yes | Yes | Yes | Yes | Not applicable |
| Cunha et al., 2022 | Yes | Yes | Yes | Yes | Yes | Yes | Not applicable |
| Druke et al., 2021 | Yes | NO | Yes | Yes | Yes | Yes | Not applicable |
| Elhusseiny et al., 2022 | Yes | Yes | Yes | Yes | Yes | Yes | Not applicable |
| ElSheikh et al., 2021 | Yes | Yes | Yes | Yes | Yes | Yes | Not applicable |
| Endo et al., 2021 | Yes | Yes | Yes | Yes | Yes | Yes | Not applicable |
| Fard et al., 2022 | Yes | Yes | Yes | Yes | Yes | Yes | Not applicable |
| Fard et al., 2022 | Yes | Yes | Yes | Yes | Yes | Yes | Not applicable |
| Forshaw et al., 2022 | Yes | Yes | Yes | Yes | Yes | Yes | Not applicable |
| Gabrielle et al., 2022 | Yes | Yes | Yes | Yes | NO | NO | Not applicable |
| Goyal et al., 2021 | Yes | NO | Yes | Yes | Unclear | Yes | Not applicable |
| Grunenwald et al., 2022 | Yes | Yes | Yes | Yes | Yes | Yes | Not applicable |
| Hasegawa et al., 2022 | Yes | Yes | Yes | Yes | Yes | Yes | Not applicable |
| Hwang et al., 2022 | Yes | Yes | Yes | Yes | Yes | Yes | Not applicable |
| Inagawa et al., 2022 | Yes | NO | Yes | Yes | Yes | Yes | Not applicable |
| Iwai et al., 2021 | Yes | Yes | Yes | Yes | Yes | Yes | Not applicable |
| Jalink et al., 2022 | Yes | NO | Yes | Yes | Yes | Yes | Not applicable |
| Jumroendararasame et al., 2021 | Yes | Yes | Yes | Yes | NO | Yes | Not applicable |
| Kang et al., 2022 | Yes | Yes | Yes | Yes | Yes | Yes | Not applicable |
| Khochtali et al., 2021 | Yes | NO | Yes | Yes | Yes | Yes | Not applicable |
| Kim et al., 2022 | Yes | Yes | Yes | Yes | Yes | Yes | Not applicable |
| Leber et al., 2021 | No | NO | Yes | Yes | Yes | NO | Not applicable |
| Lee et al., 2022 | Yes | Yes | Yes | Yes | Yes | Yes | Not applicable |
| Lee et al., 2022 | Yes | Yes | Yes | Yes | Yes | Unclear | Not applicable |
| Lee et al., 2022 | Yes | NO | Yes | Yes | Yes | Yes | Not applicable |
| Lin et al., 2022 | Yes | NO | Yes | Yes | Yes | Yes | Not applicable |
| Lin et al., 2022 | Yes | Yes | Yes | Yes | Yes | Yes | Not applicable |
| Liu et al., 2022 | Yes | Yes | Yes | Yes | Yes | Yes | Not applicable |
| Lo et al., 2022 | Yes | Yes | Yes | Yes | Yes | Yes | Not applicable |
| Maleki et al., 2021 | Yes | Yes | Yes | Yes | Yes | Yes | Not applicable |
| Maleki et al., 2021 | Yes | Yes | Yes | Yes | Unclear | Yes | Not applicable |
| Mambretti et al., 2021 | Yes | Yes | Yes | Yes | NO | NO | Not applicable |
| Mambretti et al., 2021 | Yes | Yes | Yes | Yes | NO | NO | Not applicable |
| Mohammadzadeh et al., 2022 | Yes | Yes | Yes | Yes | Yes | Yes | Not applicable |
| Mohammadzadeh et al., 2022 | Yes | Yes | Yes | Yes | Yes | Yes | Not applicable |
| Nagaratnam et al., 2022 | Yes | Yes | Yes | Yes | Yes | Yes | Not applicable |
| Nahata et al., 2022 | Yes | Yes | Yes | Yes | Yes | Yes | Not applicable |
| Ninet et al., 2022 | Yes | Yes | Yes | Yes | NO | Yes | Not applicable |
| Pan et al., 2021 | Yes | Yes | Yes | Yes | Yes | Yes | Not applicable |
| Pur et al., 2022 | Yes | Yes | Yes | Yes | NO | Yes | Not applicable |
| Rallis et al., 2021 | Yes | Yes | Yes | Yes | Yes | Yes | Not applicable |
| Renisi et al., 2021 | Yes | Yes | Yes | Yes | Yes | Yes | Not applicable |
| Rennie et al., 2022 | Yes | Yes | Yes | Yes | Yes | NO | Yes |
| Reshef et al., 2022 | Yes | Yes | Yes | Yes | Yes | Yes | Not applicable |
| Reshef et al., 2022 | Yes | Yes | Yes | Yes | Yes | Yes | Not applicable |
| Reshef et al., 2022 | Yes | Yes | Yes | Yes | Yes | Yes | Not applicable |
| Richardson et al., 2021 | Yes | Yes | Yes | Yes | Yes | Yes | Not applicable |
| Ryu et al., 2022 | Yes | Yes | Yes | Yes | Yes | Yes | Not applicable |
| Ryu et al., 2022 | Yes | Yes | Yes | Yes | Not clear | Yes | Not applicable |
| Sacconi et al., 2022 | Yes | Yes | Yes | Yes | Not clear | Yes | Not applicable |
| Salai et al., 2022 | Yes | Yes | Yes | Yes | Yes | Yes | Not applicable |
| Sanjay et al., 2022 | Yes | Yes | Yes | Yes | Yes | Yes | Not applicable |
| Sanjay et al., 2022 | Yes | Yes | Yes | Yes | Yes | Yes | Not applicable |
| Santovito et al., 2021 | Not clear | NO | Yes | Yes | Yes | Yes | Not applicable |
| Sarigul Sezenoz et al., 2022 | Yes | Yes | Yes | Yes | NO | Yes | Not applicable |
| Savino et al., 2022 | Yes | NO | Yes | Yes | Yes | Yes | Not applicable |
| Savino et al., 2022 | Yes | Yes | Yes | Yes | Yes | Yes | Not applicable |
| Savino et al., 2022 | Yes | Yes | Yes | Yes | Yes | Yes | Not applicable |
| Sezenoz et al., 2022 | Yes | NO | Yes | Yes | Yes | Yes | Not applicable |
| Shah et al., 2021 | Yes | NO | Yes | Yes | Yes | Yes | Not applicable |
| Shah et al., 2021 | Yes | Yes | Yes | Yes | Yes | Yes | Not applicable |
| Shah et al., 2022 | Yes | Yes | Yes | Yes | Yes | Yes | Not applicable |
| Smith et al., 2022 | Yes | Yes | Yes | Yes | Not clear | Yes | Not applicable |
| Sodhi et al., 2022 | Yes | Yes | Yes | Yes | Yes | Yes | Not applicable |
| Sonmez et al., 2021 | Yes | Yes | Yes | Yes | Yes | Yes | Not applicable |
| Subramony et al., 2021 | Yes | Yes | Yes | Yes | Yes | Yes | Not applicable |
| Sugihara et al., 2022 | Yes | Yes | Yes | Yes | Yes | Yes | Not applicable |
| Subramony et al., 2021 | Yes | Yes | Yes | Yes | Yes | Yes | Not applicable |
| Sung et al., 2023 | Yes | Yes | Yes | Yes | Yes | Yes | Not applicable |
| Tanaka et al., 2021 | Yes | Yes | Yes | Yes | Yes | Not clear | Not applicable |
| Tanaka et al., 2021 | Yes | Yes | Yes | Yes | Yes | Yes | Not applicable |
| Tomishige et al., 2022 | Yes | NO | Yes | Yes | Yes | Yes | Not applicable |
| Tsukii et al., 2021 | Yes | Yes | Yes | Yes | Yes | Yes | Not applicable |
| Valenzuela et al., 2021 | Yes | Yes | Yes | Yes | Yes | Yes | Not applicable |
| Valsero Franco et al., 2022 | Yes | Not clear | Yes | Yes | Yes | Yes | Not applicable |
| Valsero Franco et al., 2022 | Yes | Not clear | Yes | Yes | Yes | Yes | Not applicable |
| Vinzamuri et al., 2021 | Yes | NO | Yes | Yes | NO | Yes | Not applicable |
| Wang et al., 2022 | Yes | Yes | Yes | Yes | Yes | Yes | Not applicable |
| Wang et al., 2022 | Yes | Yes | Yes | Yes | Yes | Yes | Not applicable |
| Wasser et al., 2021 | Yes | Yes | Yes | Yes | Yes | Yes | Not applicable |
| Wasser et al., 2021 | Yes | Yes | Yes | Yes | Yes | Yes | Not applicable |
| Yamaguchi et al., 2022 | Yes | NO | Yes | Yes | Yes | Yes | Not applicable |
| Yasuda et al., 2022 | Yes | Yes | Yes | Yes | Yes | Yes | Not applicable |
| Yucel Gencoglu et al., 2022 | Yes | Yes | Yes | Yes | Yes | Yes | Not applicable |
| Zheng et al., 2021 | Yes | Yes | Yes | Yes | Yes | NO | Not applicable |
